# RNA sequencing of whole blood in dogs with primary immune-mediated hemolytic anemia (IMHA) reveals novel insights into disease pathogenesis

**DOI:** 10.1371/journal.pone.0240975

**Published:** 2020-10-22

**Authors:** Corie Borchert, Adam Herman, Megan Roth, Aimee C. Brooks, Steven G. Friedenberg

**Affiliations:** 1 Department of Veterinary Clinical Sciences, University of Minnesota College of Veterinary Medicine, St. Paul, Minnesota, United States of America; 2 Minnesota Supercomputing Institute, University of Minnesota, Minneapolis, Minnesota, United States of America; 3 Department of Veterinary Clinical Sciences, Purdue University College of Veterinary Medicine, West Lafayette, Indiana, United States of America; Colorado State University, UNITED STATES

## Abstract

Immune-mediated hemolytic anemia (IMHA) is a life-threatening autoimmune disorder characterized by a self-mediated attack on circulating red blood cells. The disease occurs naturally in both dogs and humans, but is significantly more prevalent in dogs. Because of its shared features across species, dogs offer a naturally occurring model for studying IMHA in people. In this study, we used RNA sequencing of whole blood from treatment-naïve dogs to study transcriptome-wide changes in gene expression in newly diagnosed animals compared to healthy controls. We found many overexpressed genes in pathways related to neutrophil function, coagulation, and hematopoiesis. In particular, the most highly overexpressed gene in cases was a phospholipase scramblase, which mediates the externalization of phosphatidylserine from the inner to the outer leaflet of cell membranes. This family of genes has been shown to be critically important for programmed cell death of erythrocytes as well as the initiation of the clotting cascade. Unexpectedly, we found marked underexpression of many genes related to lymphocyte function. We also identified groups of genes that are highly associated with the inflammatory response and red blood cell regeneration in affected dogs. We did not find any genes that distinguished dogs that lived vs. those that died at 30 days following diagnosis, nor did we find any relevant genomic signatures of microbial organisms in the blood of affected animals. Future studies are warranted to validate these findings and assess their implication in developing novel therapeutic approaches for dogs and humans with IMHA.

## Introduction

Immune-mediated hemolytic anemia (IMHA) is an autoimmune disorder characterized by an endogenous immune system attack against erythrocyte surface antigens and subsequent red blood cell lysis [1]. The disease occurs naturally in both humans and dogs and shares similar features across both species [2, 3], but is significantly more common in dogs. Epidemiologic studies have shown that the incidence of IMHA is 1–3 per 100,000 patients annually in humans [3], but greater than 1 in 10,000 in certain dog breeds [2, 4]. Dogs therefore present a naturally occurring model for studying the pathogenesis of a rare human disease with significant morbidity.

In humans, the disease is commonly referred to as autoimmune hemolytic anemia (AIHA) as the self-mediated attack on erythrocytes has been well characterized [3, 5]. AIHA is categorized into warm antibody types (primary and secondary warm antibody AIHA), cold antibody types (primary cold agglutinin disease, secondary cold agglutinin syndrome, paroxysmal cold hemoglobinuria), and atypical AIHA (mixed warm and cold-agglutinin, direct antiglobulin test negative AIHA) [5]. The warm antibody type accounts for 75% of human AIHA cases [3]. In all forms of the disease, IgG and IgM autoantibodies are deposited on erythrocyte cell membranes, leading to complement activation and subsequent hemolysis. The proposed disease mechanism involves impaired distinction of self and non-self with inadequate T cell-mediated regulation of the humoral immune system; this in turn leads to activation of macrophages and complement, followed by erythrocyte lysis [5]. The underlying cause of AIHA remains poorly understood, however a genetic predisposition has been suggested in some cases [5–7]. Treatment for AIHA typically involves steroid therapy, secondary immunosuppressive agents, intravenous immunoglobulin, or plasmapheresis, with splenectomy often recommended in refractory cases [3].

In dogs, IMHA shares similar warm and cold antibody types, with the vast majority of cases being the warm antibody type [8]. Veterinarians therefore typically classify IMHA cases into primary (non-associative) vs. secondary (associative) types. Primary IMHA is defined as a type II hypersensitivity reaction with no identifiable cause that results in the production of immunoglobulins against erythrocyte surface antigens, followed by intravascular complement-mediated destruction and/or extravascular mononuclear cell destruction through opsonization and phagocytosis [9]. Secondary IMHA occurs when the disease can be attributed to an identifiable underlying etiology [1]. Primary IMHA comprises upwards of 75% of cases [10]. A genetic link has been suggested in several breeds, including Cocker Spaniels and Springer Spaniels, where odds ratios of developing IMHA are greater than 10 for both breeds [2, 4]. Diagnosis is based upon a combination of several laboratory values and consensus guidelines for diagnosing IMHA have recently been proposed [1]. Treatment involves the use of similar immunosuppressive agents as in humans along with anti-thrombotic agents [11]. Plasmapheresis and splenectomy have also been described as treatment options in dogs [12, 13]. Mortality rates for dogs with IMHA are relatively high compared to humans, ranging from 21–83% according to several studies [14–22].

In general, the immunologic, cellular, and genetic mechanisms that lead to IMHA are poorly understood in dogs. As in humans, studies have documented the presence of erythrocyte surface IgG and IgM in dogs with IMHA [23], however the primary antigenic targets in dogs have not been established conclusively [24, 25]. Upregulation of specific cytokines including tumor necrosis factor ɑ (TNFɑ) and C-X-C motif chemokine ligand 8 (CXCL8) has been reported in dogs [9], however an exhaustive characterization of the immunologic response has never been performed. While several studies have shown an association between class II dog leukocyte antigen alleles and IMHA [26], which parallels associations in humans [27, 28], no genome-wide analysis of variants associated with IMHA has been published in dogs. Gaining a better understanding of the pathophysiology of IMHA may lead to new treatment options and better clinical outcomes for canine patients, and improve the usefulness of the dog as a model for the analogous human disease.

Transcriptome-wide gene expression studies have been performed for many diseases in both human and veterinary medicine [29–32]. These studies have proven powerful in informing disease pathophysiology, identifying causative genes, and discovering novel therapeutic targets [33–35]. In this study, we used RNA sequencing to improve our understanding of canine IMHA. Our primary objective was to compare gene expression differences in the blood of treatment-naive, newly diagnosed dogs with IMHA compared to healthy control dogs. We hypothesized that genes and gene pathways related to the adaptive immune system response would be markedly altered in affected animals, providing insights into the underlying disease mechanisms and potentially elucidating novel targets for therapeutic intervention.

## Materials and methods

### Study population and sample collection

We recruited client-owned dogs with a new diagnosis of IMHA presenting to the Emergency Services at the University of Minnesota Veterinary Medical Center (approved IACUC protocol 1606-33196A) and the Purdue University Veterinary Teaching Hospital (approved IACUC protocol 1510001313). Informed, written consent was obtained from each dog’s owner prior to enrollment in the study. All dogs were required to have had a complete blood count (CBC) with manual differential and chemistry profile performed at a reference laboratory on presentation. Inclusion criteria were as follows: (1) packed cell volume <30%; (2) spherocytosis, a positive saline agglutination test, or a positive Coomb’s test; and (3) hyperbilirubinemia, hemoglobinuria, or erythrocyte ghost cells. Animals were excluded if they met any of the following conditions: (1) current or prior treatment for IMHA or its sequelae (e.g., steroids, other immunosuppressive therapy, thromboprophylaxis, blood transfusion, fluid therapy); (2) current or recent (past 30 days) use of any medications except for flea, heartworm, and tick prevention; (3) evidence of secondary immune-mediated disease (e.g., neoplasia, tick-borne disease) based upon imaging studies or infectious disease serology/PCR testing; (4) less than one year of age. For each case, we recorded breed, age, sex, submitting institution, relevant laboratory values, survival to discharge, and 30-day survival.

We also enrolled apparently healthy, breed-, sex-, and age-matched (±2 years) control dogs for each of our cases. Control dogs were recruited through the Primary Care Service at the University of Minnesota Veterinary Medical Center. Dogs were required to have presented for a routine wellness appointment and have had an unremarkable physical examination performed by a veterinarian. All control dogs had a CBC with manual differential performed at a reference laboratory upon enrollment. Exclusion criteria were as follows: (1) use of any medications except for flea, heartworm, and tick prevention in the past 30 days; (2) vaccination within the past 30 days; (3) less than one year of age. For each control dog, we recorded breed, age, sex, and relevant laboratory values.

We collected 2–3 mL of whole blood from each dog in a Tempus Blood RNA tube (ThermoFisher Scientific, Waltham, MA). For affected dogs, blood samples were collected prior to any therapeutic intervention. Following blood collection, an animal’s participation in the study ended and all further medical decisions were made by the attending veterinarian. For samples collected at the University of Minnesota, blood was held in storage overnight at 4°C or transferred to our laboratory immediately depending upon the time of day in which the sample was obtained. Once in the laboratory, RNA-preserved blood was aliquoted and stored at -80°C for further processing. For samples collected at Purdue University, blood was stored at 4°C until the next business day, at which point samples were shipped overnight on ice to the University of Minnesota; upon arrival, RNA-preserved blood was aliquoted and stored at -80°C for further processing. In all cases, blood samples were stored and processed according to the manufacturer’s guidelines for the use of Tempus Blood RNA tubes.

### RNA processing, library preparation, and sequencing

RNA was isolated from thawed RNA-preserved blood using the Tempus Spin RNA Isolation Kit (ThermoFisher Scientific, Waltham, MA) following the manufacturer’s instructions. Samples were analyzed on a Nanodrop 8000 spectrophotometer (ThermoFisher Scientific, Waltham, MA) and an Agilent 2100 Bioanalyzer (Agilent Technologies, Santa Clara, CA) to measure RNA concentration and integrity, respectively. Samples with an RNA Integrity Number (RIN) < 7 were excluded from downstream analysis.

Approximately 500 ng of RNA from each dog was submitted for library preparation and sequencing at the University of Minnesota Genomics Center. Library preparation and dual-indexed barcoding was performed using the Illumina TruSeq Stranded Total RNA with Ribo-Zero Globin kit (Illumina, San Diego, CA) following the manufacturer’s protocols. This library preparation method captures total RNA and then depletes ribosomal RNA and hemoglobin (Hb) RNA using sequence-specific capture probes. These probes are compatible with canine RNA [36]. Library size distribution was validated using capillary electrophoresis and quantified using the Quant-iT RiboGreen RNA Assay kit (ThermoFisher Scientific, Waltham, MA). Indexed libraries were normalized, pooled, and size-selected to 320 bp ± 5% using a Caliper LabChip XT (PerkinElmer, Waltham, MA) to generate libraries with mean insert sizes of 200 bp.

Pooled libraries were sequenced in one lane of a NovaSeq 6000 System (Illumina, San Diego, CA) using an S2 flow cell in a 50-base-pair, paired-end read configuration. Base calls were made using Real Time Analysis software (Illumina, San Diego, CA). Samples were de-multiplexed and converted to FASTQ format using bcl2fastq 2.20 (Illumina, San Diego, CA). Quality assurance of FASTQ reads was performed using FastQC 0.11.7 [37]. FASTQ files have been made publicly available through NCBI’s Short Read Archive (temporary reviewer link: https://dataview.ncbi.nlm.nih.gov/object/PRJNA629466?reviewer=tql0m7t6qgp4c5pem7h0ad0006, link after publication: https://www.ncbi.nlm.nih.gov/sra/PRJNA629466).

### Estimation of RNA abundance and fraction by cell type

We collected published reference values of RNA per cell for different peripheral blood cell types from studies in humans and rabbits (reticulocytes only, as human reticulocyte data was not available). We then multiplied RNA per cell type by cell counts from our CBC data to estimate the RNA abundance by cell type in each dog. Published values in picograms (pg) of total RNA per cell are as follows: neutrophils 0.0286 [38], monocytes 0.751 [38], lymphocytes 0.435 [38], eosinophils 0.0494 [38], RBCs 0.000570 [39], platelets 0.00220 [39], and reticulocytes 0.104 [40] (NB: Reticulocyte RNA per cell was calculated from the cited manuscript assuming a mean corpuscular volume of 62 fL in rabbits [41]). Because our library preparation included Hb depletion and Hb transcripts represent ~70% of RBC RNA [42] and ~95% of reticulocyte RNA [43], we assumed that RBCs contributed 0.000171 pg/cell and reticulocytes contributed 0.00518 pg/cell after Hb depletion. For each dog, we converted total RNA by cell type into a fraction, applied a log_2_ transformation, and estimated the log_2_ fold-change (log_2_FC) and 95% confidence interval in RNA fraction by cell type between cases and controls. Calculations were performed in R 3.6.2 [44].

### Bioinformatics

#### Differential gene expression

Raw FASTQ reads were pseudo-mapped to the Ensembl 98 CanFam 3.1 reference transcriptome [45] using Salmon 1.0 [46] with default settings. Salmon output was imported into R 3.6.2 [44] using tximport 1.14.2 [47] to summarize transcript-level abundance, counts, and lengths for gene-level analysis. Analysis of differentially expressed genes (DEGs) was performed using DESeq2 1.26.0 [48] with default settings; only transcripts with read counts ≥ 10 in at least 5 samples were included in DEG testing. Principal component analysis (PCA) was performed for sample outlier detection using the “prcomp()” and “pairs()” functions in R 3.6.2 with variance stabilizing transformation (VST) transformed read counts as inputs. Population comparisons for DEGs included cases vs. controls, and among cases, alive vs. dead at 30 days. DEGs were considered significant if they met all of the following conditions: Benjamini-Hochberg (BH)-corrected p-value ≤ 0.05, |log_2_FC| between groups ≥ 2, mean of normalized counts across samples (baseMean) ≥ 10. Use of an |log_2_FC| ≥ 2 cutoff was based on our estimation of the change in RNA abundance by cell type between cases and controls (see Results section below). DEGs with an |log_2_FC| ≥ 3 were also individually researched to understand their potential role in the pathogenesis of IMHA. A heatmap of significant DEGs using VST-transformed read counts was created using the pheatmap package in R [49]. Conversion of Ensembl IDs to gene symbols and descriptions was performed using BioMart [50, 51] implemented in R; some gene symbols were identified manually using Ensembl human or mouse orthologues.

#### Pathway analysis

Significant DEGs were sorted by log_2_FC and pathways analysis was performed using Gene Set Enrichment Analysis (GSEA) 4.0.3 [52, 53] in pre-ranked mode following best practices described in the literature [54]. In order to minimize overlapping results, we limited our analysis to the following Molecular Signatures Database (MSigDB) 7.1 collections: hallmark gene sets (H), curated gene sets (C2) canonical and Reactome pathways, and Gene Ontology (GO) gene sets (C5) biological process pathways [52, 55, 56]. Pathways with a BH-corrected p-value ≤ 0.1 were considered significant, in keeping with the exploratory nature of this work as well as recommendations made by others [54]. Single-sample GSEA 10.0.2 [52] using transcript-per-million (TPM) inputs [57] calculated by Salmon and tximport was performed in GenePattern 2.0 [58] to aggregate expression of individual genes into a single enrichment score per dog per pathway. We used the EnrichmentMap plugin [59] in Cytoscape 3.7.2 [60] to visualize and organize significant pathways using an edge cutoff (similarity score) of 0.375, again following best-practice guidelines [54]. Pathways were filtered to display nodes with at least two neighbors to simplify visualization.

#### Gene correlation network analysis

We used the Weighted Correlated Network Analysis (WGCNA) R package 1.69 [61, 62] to identify clusters of highly correlated genes (referred to as “modules”). Using VST-transformed read counts of genes with baseMean ≥ 10 as inputs [63], we first determined the appropriate soft-thresholding power (in our case, 17) for calculating the expression adjacency matrix, which was then used as input for calculating the topological overlap matrix. The topological overlap matrix is the primary analysis unit in WGCNA, and with it we created expression modules that were merged based on a correlation cutoff of >30%. We then used WGCNA to test the relationship of these modules with measured blood cell counts (referred to as “traits”) for RBCs, reticulocytes, neutrophils, monocytes, lymphocytes, eosinophils, and platelets in our patient population.

We further explored four significant module-trait relationships based upon their importance in canine IMHA in order to identify biologically relevant genes whose expression is correlated with counts of these blood cell types. For each module-trait relationship, genes within each module were selected that met the following conditions: p-value for gene significance (GS) ≤ 0.05, p-value for module membership (MM) ≤ 0.05, MM ≥ 0. (NB: GS represents the correlation between gene expression and a trait, and MM represents the correlation between gene expression and a module [61, 62]). Selected genes were evaluated for overrepresentation in annotated gene sets using Enrichr [64, 65]. Gene sets with biological relevance to the trait being evaluated (e.g., the gene set “neutrophil activation involved in immune response” for the trait “neutrophil count”) and statistically significant Enrichr combined scores [64] were searched for overlap with the input gene list. Expression of overlapping genes was examined relative to cell counts for each trait. Continuous trait data was collapsed into clinically relevant groupings (e.g., above reference range vs. reference range, or quantiles for traits where no reference range is available) and gene expression heatmaps with clustering were created using pheatmap [49]. Differences in gene expression for individual genes across groupings using TPM values as inputs were evaluated using a one-way ANOVA followed by a Tukey’s range test, with p-values ≤ 0.05 considered significant. Statistical calculations were performed in R 3.6.2 [44].

#### Case cluster analysis

Hierarchical clustering of gene expression data was used to organize affected dogs into groups with potential clinical significance. VST-transformed read counts were used to calculate a Euclidean distance matrix, and clustering was performed using the hclust function in R [44] using Ward’s revised agglomeration method [66]. We used the Sum of Squared Error (SSE) method to determine an appropriate number of clusters for downstream analysis [67]. Based upon this output, dogs were assigned to case clusters which were used as categorical traits in WGCNA to identify modules correlated with these clusters. Genes within highly correlated modules were selected as described above (p-value for GS ≤ 0.05, p-value for MM ≤ 0.05, MM ≥ 0) and tested for differences in expression between cluster groups using a t test, with an FDR p-value ≤ 0.05 considered significant. Significantly different genes were evaluated for overrepresentation using Enrichr [64, 65], and pathways with the highest combined scores [64] were searched for overlap with the input gene list. Expression of overlapping genes was converted to log_2_FC format to visualize differences in gene expression between case clusters. Statistical calculations were performed in R 3.6.2 [44].

#### Analysis of unmapped reads for microbial signatures

Raw FASTQ reads were trimmed using Trimmomatic 0.33 [68], mapped to the Ensembl 98 CanFam 3.1 reference genome [45] using HISAT 2.1.0 [69] with default settings, and converted to sorted BAM files using samtoools 1.7 [70]. BAM file quality assurance was performed using Picardtools 2.9 [71] and RNASeQC 2.35 [72]. We identified unmapped reads from BAM files and extracted them from the original FASTQs using seqkit 0.8.1 [73]. Unmapped reads were evaluated using an in-house bioinformatics pipeline called shotmeta [74], which includes read classification with Kraken 1.0 [75] and species-specific assignment using Bracken [76]. The Kraken 1.0 database includes the complete virus, phage, plasmid, mitochondrion, bacteria, fungi, protozoan and human databases from RefSeq [77] as well as all viral and phage samples in GenBank [78] as of December 3, 2019.

Species-level abundances were summarized as read counts, normalized by library size, and used as predictors in a least absolute shrinkage and selection operator (LASSO) model with disease status as the response variable. We performed stability selection of predictor variables using the “stabsel()” function in the stabs R package 0.6.3 [79, 80], which consisted of running 100 LASSO models with different subsets of the data with the expectation that relevant taxa will remain predictors in a majority of the runs. Because this analysis was exploratory, we used a lenient frequency cutoff of 60% and a per-family error rate (PFER, a type of multiplicity correction) of two for determining stable predictors of IMHA disease status [79]. Adjusting the frequency cutoff to 75% and the PFER between 1 and 2 had no significant effect on the results.

Unmapped reads for specific taxa were assembled using Geneious Prime 2020.1.2 (Biomatters Ltd., Auckland, New Zealand) and further analyzed using nucleotide basic local alignment search tool (BLASTn, [81]).

### Quantitative real-time PCR

We performed relative quantitative PCR (qPCR) on RNA for five genes in the “GO T cell differentiation” pathway from six affected (D02390, D02639, D03309, D04594, D04596, D04672) and four unaffected (D03129, D04676, D04772, D04933) dogs whose lymphocyte counts at the time of blood collection were ~1 × 10^3^/μL. This pathway was the most highly downregulated pathway in our dataset. Genes evaluated included CCR7, CD80, GATA3, IL2, and ITK. Housekeeping genes included GAPDH, which is commonly used as a standard in relative qPCR experiments [82], as well as HEATR5B and SCFD1. The latter two genes were selected from our RNA-seq data using methods recommended in the literature [83–85]. Briefly, using VST-transformed read counts, we selected genes with the lowest coefficient of variation (~1) with baseMean counts between 500 and 3,000 where the |log_2_FC| in expression between affected and unaffected dogs was < 0.1.

cDNA was synthesized from 1.5 μg of RNA per dog using the BioRad iScript cDNA Synthesis Kit, and qPCR was performed with BioRad iTaq™ Universal SYBR® Green Supermix, following the manufacturer’s protocols (BioRad, Hercules, CA). Reactions contained 1 μl cDNA and 500 nM gene-specific primers (S1 Table), and were performed in triplicate. Relative quantitation of each pathway gene compared to each housekeeping gene was performed using the ΔΔC_T_ method [86].

## Results

### Animals

We enrolled 21 affected and 18 unaffected dogs; three unaffected dogs served as controls for two cases each. The most common dog breed was mixed breed and the median age at presentation was 7 years (range 1–15 years). Thirteen cases were recruited from the University of Minnesota and 8 from Purdue University. All dogs met the “diagnostic for” or “supportive of” criteria for diagnosing IMHA according to the ACVIM consensus guidelines, which were published after we had started enrolling cases [1]. Patient data for cases is shown in Table 1 and S2 Table; complete patient data for controls is shown in S2 Table.

**Table 1:** Selected demographic and laboratory values for enrolled cases.

Dog ID	Age (years)	Breed	Sex	Institution	PCV (%)	Reticulocytes (× 10^3^/μL)	Spherocytes	SAT	Coomb’s Test	Serum Bilirubin (mg/dL)	Hemoglobinuria	Ghost Cells	ACVIM Consensus Classification
**D02111**	3	Collie	F	Minnesota	14%	295	Y	+	NA	14.0	Y	N	diagnostic
**D02177**	10	Pekingese	M	Minnesota	14%	54	Y	+	NA	0.6	NA	N	diagnostic
**D02390**	2	Miniature Pinscher	F	Minnesota	24%	139	Y	+	NA	2.7	Y	N	diagnostic
**D03024**	7	Shih Tzu	F	Purdue	8%	206	Y	+	NA	NA	NA	Y	diagnostic
**D03309**	6	Mixed	M	Minnesota	23%	125	Y	-	NA	1.9	Y	N	supportive
**D04932**	9	Australian Shepherd	F	Purdue	21%	461	Y	+	NA	1.6	NA	N	diagnostic
**D02251**	9	Labrador Retriever	F	Minnesota	20%	523	Y	+	NA	0.9	NA	N	diagnostic
**D02286**	7	Dachshund	M	Minnesota	15%	508	Y	-	NA	1.3	NA	N	supportive
**D02639**	8	Dachshund	F	Minnesota	15%	199	Y	-	+	0.8	N	N	diagnostic
**D03441**	11	Pekingese	F	Minnesota	13%	116	Y	+	NA	0.4	N	N	diagnostic
**D04123**	10	Rottweiler	F	Minnesota	13%	185	Y	+	NA	0.9	NA	N	diagnostic
**D04596**	15	Shih Tzu	F	Minnesota	27%	180	Y	+	NA	0.4	NA	N	diagnostic
**D04672**	11	Rat Terrier	F	Minnesota	9%	180	N	+	NA	4.6	Y	N	supportive
**D02343**	7	Mixed	M	Purdue	27%	146	Y	-	NA	2.1	N	N	supportive
**D02880**	7	Boxer	M	Minnesota	8%	470	Y	+	NA	1.3	NA	N	diagnostic
**D04274**	7	Mixed	F	Purdue	28%	83	Y	+	NA	2.2	Y	N	diagnostic
**D04594**	6	Mixed	F	Minnesota	27%	95	N	+	NA	0.6	NA	N	supportive
**D04603**	1	American Staffordshire Terrier	F	Purdue	18%	153	Y	+	NA	4.1	Y	N	diagnostic
**D02278**	5	Cocker Spaniel	M	Purdue	10%	6	Y	+	NA	0.5	NA	N	diagnostic
**D02357**	6	Mixed	M	Purdue	6%	5	Y	+	NA	0.7	NA	N	diagnostic
**D04629**	10	Mixed	F	Purdue	18%	86	Y	+	NA	0.1	N	N	supportive

PCV, packed cell volume; SAT, saline agglutination test; ACVIM, American College of Veterinary Internal Medicine; F, female; M, male; Y, yes; N, no; NA, not available; +, positive; -, negative.

### RNA processing and sequencing

Median RIN was 9.3 (range 7.0–9.9), and mean Phred-scaled quality scores for forward and reverse FASTQ reads were >35 for each sample. Sequencing yielded a median of 57 million reads per sample (range 49–73 million) with a median of 93.3% of reads mapping to the CanFam 3.1 genome (range 89.5–94.8%). rRNA depletion was largely successful, with a median of 0.9% of reads (range 0.2–10%) containing ribosomal RNA k-mers. Mean insert size was 170 base pairs. A median of 49% of reads mapped to known exons (range 33–60%), which is consistent with studies using total RNA library preparation methods [87]. Complete quality metrics by sample are shown in S3 Table.

### RNA abundance by cell type

Dogs with IMHA have different peripheral blood cell counts compared to healthy animals, typically characterized by elevated neutrophils and reticulocytes and decreased red blood cells (RBCs). These differences in cell counts likely influence transcript abundance using whole blood samples where RNA per library is fixed. To assist with data interpretation, we sought to estimate the extent to which gene expression might be influenced by differences in cell distribution using CBC data for each animal; this data is summarized in S1 Fig.

As expected, significant differences are present in cell counts and between cases and controls in all blood cell types except for eosinophils and lymphocytes (S1 Fig, panel A). While the vast majority of cells (>95%) in circulation in both cases and controls are comprised of RBCs, reticulocytes, and platelets (S1 Fig, panel B, left side), the estimated fraction of RNA contributed by each cell type is different between cases and controls (S1 Fig, panel B, right side). Additionally, some cell types which make up only a small fraction of peripheral blood cells by count (e.g., monocytes, lymphocytes) comprise a large fraction of estimated peripheral blood RNA. For example, our estimates suggest that on average, non-Hb RNA from reticulocytes comprised 8% of blood RNA in controls, but 27% of blood RNA in cases. Similarly, estimated RNA from lymphocytes comprised 21% of blood RNA in controls but 13% in cases.

We used RNA percentages by cell type in cases and controls to calculate estimated log_2_FC along with 95% confidence intervals (S1 Fig, panel C) in order to provide a reference for interpretation of DEGs expected to originate from particular cell types. We used this data to support our selection of a |log_2_FC| > 2 cutoff in selecting DEGs for downstream analysis, as we expected most biologically relevant changes in gene expression in IMHA to affect reticulocytes (newly generated in response to the destruction of RBCs), lymphocytes (enable and perpetuate the antibody-mediated response), and neutrophils (part of the generalized inflammatory response). Estimated log_2_FC in RNA for these cell types based upon measured changes in cellular abundance is 2.1 (95% CI 1.4 to 2.6), -0.8 (95% CI -1.4 to -0.3), and 1.3 (95% CI 0.9 to 1.7), respectively.

### Differentially expressed genes

**Fig 1:**
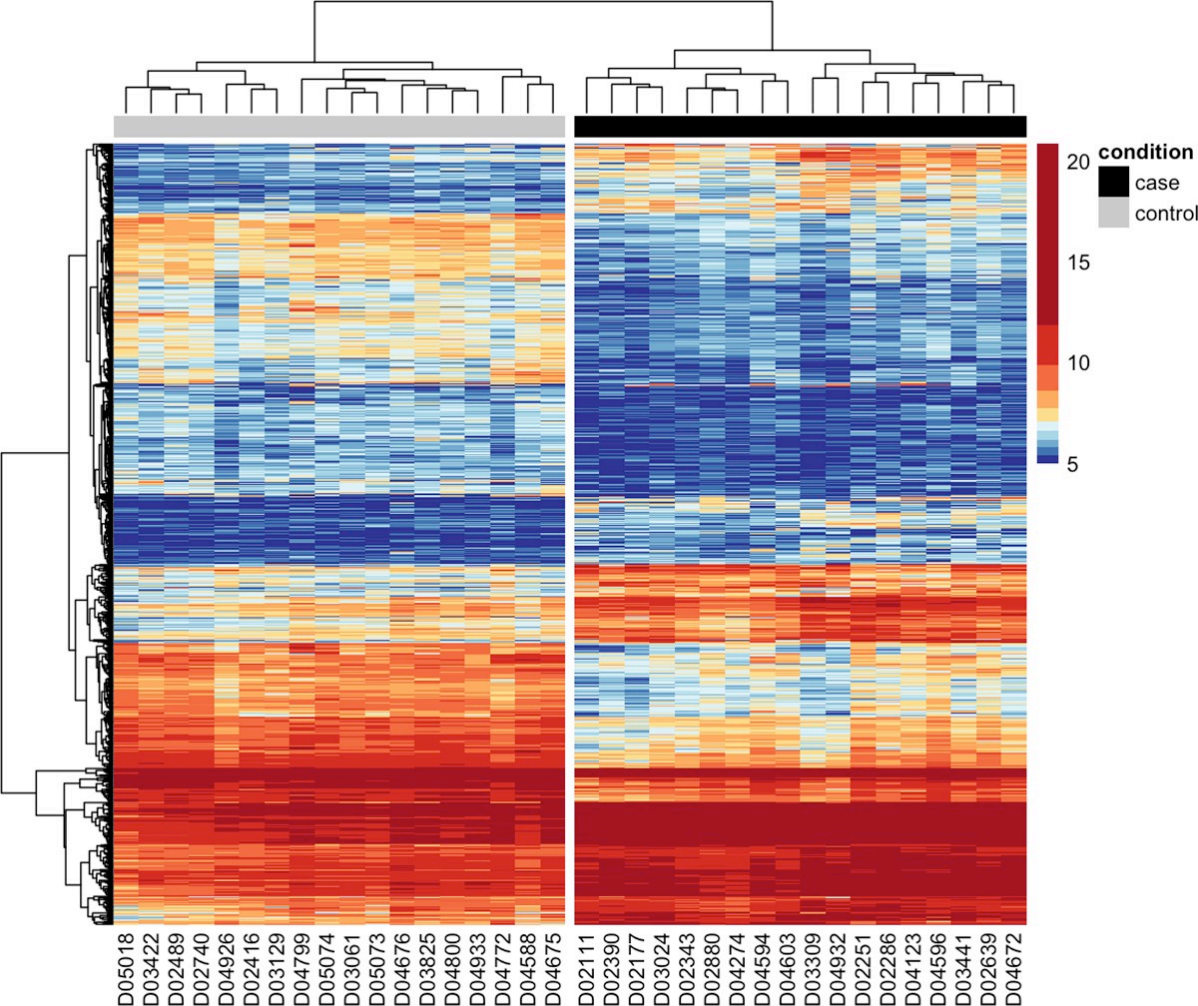
Heatmap of differentially expressed genes in dogs with IMHA vs. healthy dogs. Expression of 966 DEGs in dogs with IMHA (right) and healthy dogs (left). Gene expression values are based upon VST-transformed read counts in each dog, with higher values shown in red and lower values shown in blue.

Principal components analysis identified three case outliers (D02278, D02357, and D04629; S2 Fig), leaving 18 cases and 18 controls in our final analysis cohort. Across these samples, 14,055 genes were expressed in whole blood. Of these genes, 966 were differentially expressed in cases vs. controls (Fig 1; S4 Table); 435 were overexpressed and 531 were underexpressed in cases. The top 10 over- and underexpressed genes are shown in Table 2. No genes were differentially expressed when comparing cases alive vs. dead at 30 days following discharge.

**Table 2:** Top 10 over- and underexpressed genes in dogs with IMHA vs. healthy dogs.

Ensembl ID	Gene Symbol	Mean Expression	log_2_ Fold Change	FDR p-value	Gene Name
ENSCAFG00000031341	PLSCR5	332	8.0	2.23E-12	phospholipid scramblase family member 5
ENSCAFG00000030630	FCN2	11	6.8	4.67E-12	ficolin 2
ENSCAFG00000043810	PRR3	11	6.7	4.59E-19	proline rich 3 protein
ENSCAFG00000016149	ANXA8L1	1934	6.5	3.94E-16	annexin A8-like 1
ENSCAFG00000013807	TMEM47	47	6.3	3.39E-24	transmembrane protein 47
ENSCAFG00000029661	CFHR5	26	6.2	3.58E-20	complement factor H related 5
ENSCAFG00000008004	SBSPON	11	6.1	5.43E-17	somatomedin B and thrombospondin type 1 domain containing
ENSCAFG00000007821	YPEL4	158	5.8	6.72E-45	yippee like 4
ENSCAFG00000012292	EWSR1	35	5.8	1.86E-30	EWS RNA binding protein 1
ENSCAFG00000015272	FGFBP1	68	5.8	2.04E-18	fibroblast growth factor binding protein 1
ENSCAFG00000000255	B4GALNT1	74	-3.8	2.36E-19	beta-1,4-N-acetyl-galactosaminyltransferase 1
ENSCAFG00000013453	NKG2A	56	-3.8	1.07E-09	killer cell lectin-like receptor C1
ENSCAFG00000030133	C5H1orf141	10	-3.8	1.43E-07	chromosome 5 C1orf141 homolog
ENSCAFG00000028587	NKG2C	281	-4.1	1.60E-38	killer cell lectin-like receptor C2
ENSCAFG00000018579	GHR	32	-4.1	2.10E-15	growth hormone receptor
ENSCAFG00000011549	ELOVL6	61	-4.1	1.60E-25	fatty acid elongase 6
ENSCAFG00000049391	IGKV	47	-4.4	1.66E-08	immunoglobulin kappa variable gene segment
ENSCAFG00000031953	OLIG1	66	-5.2	1.39E-25	oligodendrocyte transcription factor 1
ENSCAFG00000043200	IGKV	38	-5.6	1.69E-05	immunoglobulin kappa variable gene segment
ENSCAFG00000020240	PMFBP1	17	-5.8	7.74E-12	polyamine modulated factor 1 binding protein 1

Mean expression shows the average normalized read counts per million across all samples; log_2_ fold change is presented as change in expression in cases vs. controls.

Because the role of all DEGs were unlikely to be explained using pathway analysis alone (see below), we researched all DEGs with a |log2FC| ≥ 3 in cases vs. controls in order to understand their potential role in the pathogenesis of IMHA. In particular, we noted that the most highly overexpressed gene in dogs with IMHA is phospholipase scramblase 5 (PLSCR5), whose expression was nearly 256-fold higher in cases vs. controls. Its paralog phospholipase scramblase 1 (PLSCR1 [LOC611500]) was over 10-fold overexpressed in cases vs. controls. This group of enzymes mediates externalization of phosphatidylserine (PS) from the inner to the outer leaflet of cell membranes, and has been shown to be critically important for programmed cell death of erythrocytes as well as the initiation of the clotting cascade [88].

We also observed increased expression of genes related to the complement cascade and the acute phase response which have been previously implicated in the pathogenesis of IMHA in dogs [16]. These include genes such as ficolin 2 (FCN2, 111-fold increased), complement component 3 (C3, 31-fold increased), complement factor H-related 5 (CFHR5, 73-fold increased), pentraxin 3 (PTX3, 32-fold increased), and cathelicidin antimicrobial peptide (CAMP, 46-fold increased). Genes encoding for many erythrocyte membrane proteins also showed increased expression in cases, including band-3 anion exchanger (SLC4A1, 18-fold increased), spectrin β chain (SPTB, 14-fold increased), and erythrocyte membrane protein band 4.2 (EPB42, 11-fold increased). Some of these genes have been previously implicated as potential autoantigens in canine IMHA [24, 25]. Additionally, the gene encoding for plasminogen activator inhibitor-1 (SERPINE1, 17-fold increased) showed elevated expression in cases; plasminogen activator inhibitor-1 (PAI-1) plays an important role antagonizing clot breakdown, and increased expression of the protein has been associated with the formation of thrombi in human patients [89]. Finally, we noted increased expression of the gene indoleamine 2,3-dioxygenase (IDO1, 50-fold increased), which catalyzes the catabolism of tryptophan and suppresses T cell responses [90]. This observation may be related to our pathway analysis findings (see next section).

### Pathway analysis

Of the 966 DEGs we identified, 734 mapped to HUGO Gene Nomenclature Committee symbols and were included in the pathway analysis. Of 892 gene sets evaluated from the MSigDB subset we described above, 580 were overexpressed, and 34 of these had an FDR p-value for enrichment ≤ 0.1; 312 were underexpressed, and 80 of these had an FDR p-value for enrichment ≤ 0.1. Complete details of altered pathways are provided in S5 Table.

**Fig 2:**
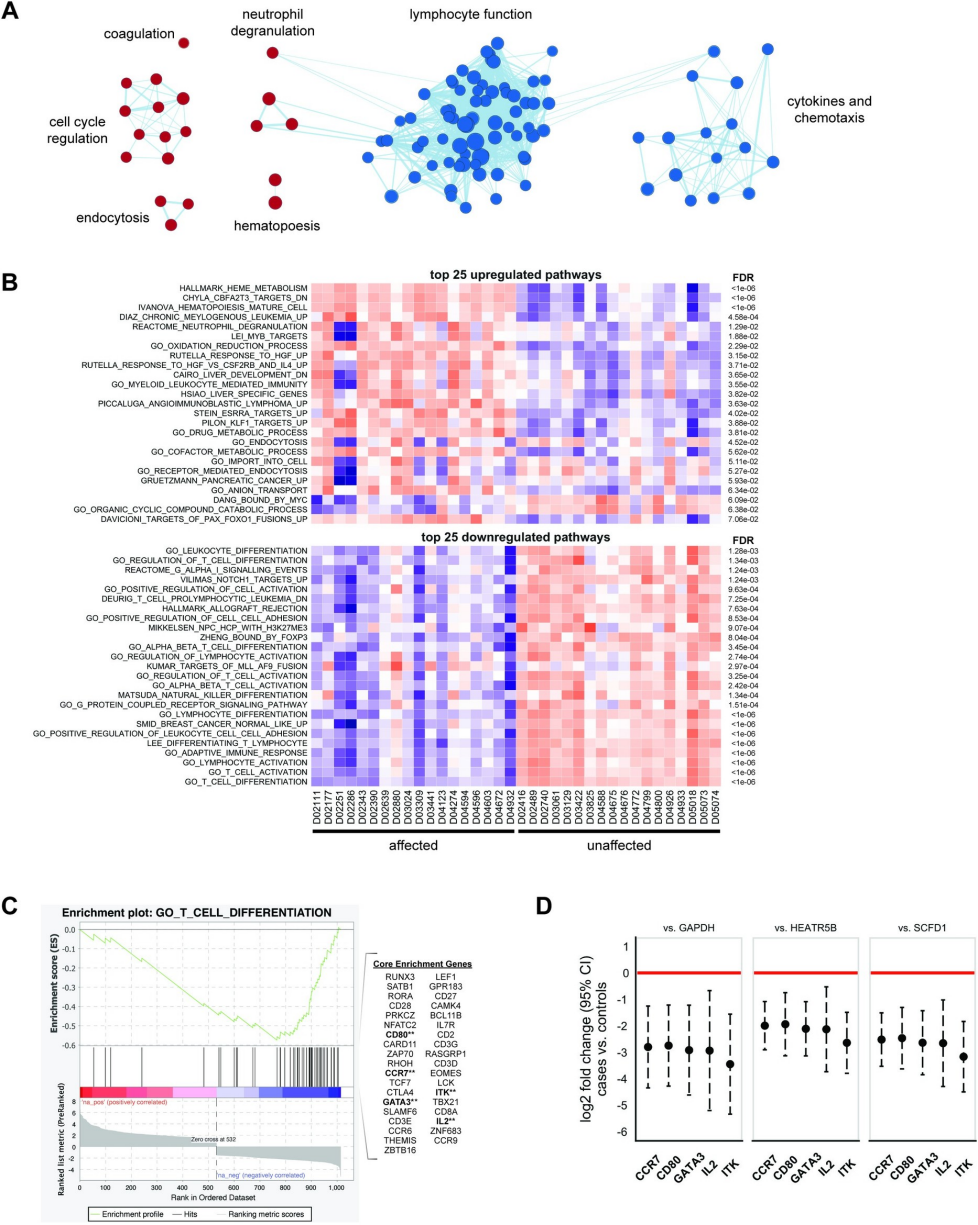
Pathway analysis. (A) Cytoscape enrichment map of over- and underexpressed GSEA pathways in dogs with IMHA. Each dot represents a GSEA pathway, and lines represent interconnectedness. Red dots represent overexpressed pathways and blue dots represent underexpressed pathways. Groupings of pathways are labeled by common activities or functions. (B) Top 25 over- and underexpressed pathways in dogs with IMHA. Overexpressed pathways are shown on the top and underexpressed pathways are shown on the bottom. A projection of enrichment of genes within each pathway from ssGSEA is shown for each dog, with affected dogs on the left and unaffected dogs on the right; red represents higher expression and blue represents lower expression. FDR p-values for pathway enrichment are shown on the right. (C) GSEA enrichment plot for “GO T cell differentiation,” which was the most underexpressed pathway in our dataset. Genes within the pathway are shown on the right, and the 5 genes evaluated by qPCR are bolded with a double asterisk. (D) Relative qPCR of 5 genes in the “GO T cell differentiation” pathway using three different housekeeping genes for normalization: GAPDH (left panel), HEATR5B (middle panel), and SCFD1 (right panel). Dots represent the average log2FC in cases vs. controls, and 95% confidence intervals are shown as dashed whiskers.

Grouping of similar pathways using EnrichmentMap in Cytoscape showed 5 groups of overexpressed pathways and 2 groups of underexpressed pathways. Overexpressed pathways included those related to neutrophil degranulation, coagulation, cell cycle regulation, endocytosis, and hematopoiesis; underexpressed pathways included those related to lymphocyte function and cytokines/chemotaxis (Fig 2A). The top 25 over- and underexpressed pathways, as well as their expression levels in individual dogs, is shown in Fig 2B. The most highly overexpressed pathway was “Hallmark heme metabolism” and the most highly underexpressed pathway was “GO T cell differentiation.”

The marked underexpression of lymphocyte-related genes was an unexpected finding, so we confirmed this underexpression in 5 genes (CD80, CCR7, GATA3, ITK, and IL2) in the most underexpressed pathway (Fig 2C). Using qPCR, we found that all 5 genes were underexpressed compared to three different housekeeping genes, consistent with our RNA-seq findings. The mean log_2_FC (95% confidence interval) in cases vs. controls relative to all housekeeping genes was as follows: CD80, -2.4 (-1.1 to -3.7); CCR7, -2.4 (-1.3 to -3.6); GATA3, -2.6 (-1.2 to -3.9); ITK, -3.1 (-1.6 to -4.5); and IL2, -2.6 (0.7 to -4.4); details are shown in Fig 2D.

### Gene networks associated with blood cell traits

Using WGCNA, we identified 11 gene modules across 13,085 expressed genes with a baseMean ≥ 10; 7 of these modules containing 7,907 expressed genes had p-values ≤ 0.05 (S3 Fig). We created a module-trait matrix using blood cell counts from our CBC data, which showed several modules highly correlated with counts of certain blood cell types (S3 Fig). In particular, the violet module (2,762 genes) was highly correlated with RBC count (r = 0.89, p = 5.7 × 10^−13^), the royal blue module (2,233 genes) was highly correlated with reticulocyte count (r = 0.68, p = 4.7 × 10^−6^), the dark turquoise module (272 genes) was highly correlated with the eosinophil count (r = 0.69, p = 2.4 × 10^−6^), and the cyan module (2,082 genes) was highly correlated with the neutrophil count (r = 0.72, p = 5.8 × 10^−5^). We explored these four module-trait relationships to identify specific genes within each module with known biological relevance to each trait, with an emphasis on neutrophils and reticulocytes given their marked alteration in dogs with IMHA.

**Fig 3:**
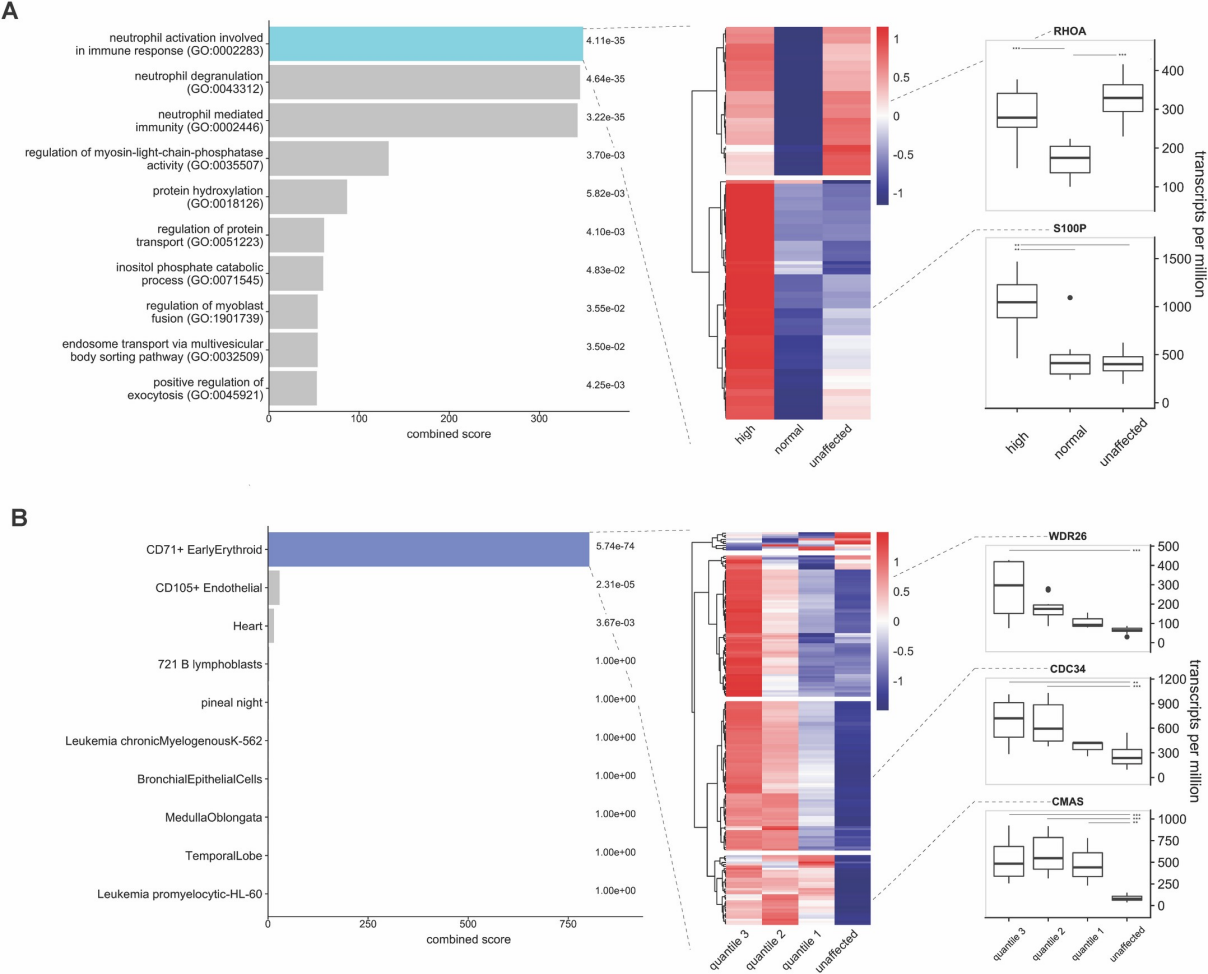
Analysis of WGCNA modules correlated with neutrophil and reticulocyte count. (A) Relationship of cyan module to neutrophil count. Left panel: Pathway analysis of 1,198 genes in the cyan module showing significant overrepresentation of three neutrophil-related pathways. Middle panel: Clustering of gene expression for genes in the top pathway in affected dogs with an elevated neutrophil count (>11.2 × 10^3^/μL; 10 dogs), affected dogs with a normal neutrophil count (8 dogs), and unaffected dogs (18 dogs). Colors show mean, centroid-scaled TPMs. Two distinct gene clusters are present, one containing genes whose expression is increased in affected dogs with an elevated neutrophil count (bottom cluster), and a second containing genes whose expression is decreased in affected dogs with a normal neutrophil count (top cluster). Right panel: Box and whiskers plots showing expression of representative genes from each cluster. (B) Relationship of royal blue module to reticulocyte count. Left panel: Pathway analysis of 1,443 genes in the royal blue module showing significant overrepresentation of an early erythroid pathway. Middle panel: Clustering of gene expression for genes in the top pathway in affected dogs with reticulocyte counts >300 × 10^6^/μL (quantile 3; 4 dogs), between 100–300 × 10^6^/μL (quantile 2; 11 dogs), below 100 × 10^6^/μL (quantile 1; 3 dogs), and unaffected dogs. Colors show mean, centroid-scaled TPMs. Three distinct gene clusters are present, one containing genes highly expressed in affected dogs in the top reticulocyte quantile (top cluster), one containing genes highly expressed in affected dogs in the top two quantiles (middle cluster), and one containing genes highly expressed in all affected dogs (bottom cluster). Right panel: Box and whiskers plots showing expression of representative genes from each cluster. For both panels, asterisks represent p-values as follows: *** ≥0.0001; ** ≥0.001; * ≥0.05.

For neutrophils, of the 2,082 genes in the cyan module, 1,198 genes met our criteria for gene significance and module membership. Evaluating these genes using Enrichr, we found three biologically relevant pathways that were significantly overrepresented: neutrophil activation involved in immune response, neutrophil degranulation, and neutrophil-mediated immunity (Fig 3A, left panel). The first of these pathways contained 115 genes in the cyan module. We evaluated the expression of these 115 genes in three groups of dogs: affected dogs with a neutrophil count above the reference range (>11.2 × 10^3^/μL; 10 dogs), affected dogs with a neutrophil count within the reference range (8 dogs), and unaffected dogs. A heatmap showing expression of these 115 genes by group (Fig 3A, middle panel) reveals two patterns of gene expression, which are shown for illustrative genes (Fig 3A, right panel). One cluster of genes is highly expressed only in affected dogs with a peripheral neutrophilia. These genes include many S100 genes, among others. A second cluster of genes shows *lower* expression in affected dogs with a normal neutrophil count compared to affected dogs with an elevated neutrophil count or healthy dogs. Examples of these genes include cell surface receptors such as integrin subunit β2 (ITGB2) and L-selectin (SELL), cytoskeleton-related proteins such as Ras homolog family member A (RHOA), and nuclear factor kappa B subunit 1 (NFKB1). Further details are provided in S6 Table.

For reticulocytes, of the 2,233 genes in the royal blue module, 1,443 genes met our criteria for gene significance and module membership. Evaluating these genes using Enrichr, we found one relevant pathway that was significantly overrepresented: CD71+ early erythroid (Fig 3B, left panel). This pathway contained 185 genes in the royal blue module. We evaluated the expression of these 185 genes in four groups of dogs: affected dogs with a reticulocyte count >300 × 10^6^/μL (quantile 3; 4 dogs), affected dogs with a reticulocyte count between 100–300 × 10^6^/μL (quantile 2; 11 dogs), affected dogs with a reticulocyte count below 100 × 10^6^/μL (quantile 1; 3 dogs), and unaffected dogs. A heatmap showing expression of these 185 genes by group (Fig 3B, middle panel) shows three patterns of gene expression, which are shown for illustrative genes (Fig 3B, right panel). One cluster of genes shows elevated expression in all affected dogs, a second cluster of genes shows elevated expression in dogs in the second and third quantiles only, and a third cluster of genes shows elevated expression only in dogs in the highest reticulocyte quantile. Unaffected dogs showed lower expression of nearly all genes within this pathway. Further details are provided in S6 Table.

Similar analyses were performed RBCs and eosinophils but without the gene clustering step. For RBCs, of the 2,762 genes in the violet module, 2,359 met our criteria for gene significance and module membership. Using Enrichr, we found 218 of these genes to be significantly overrepresented (FDR p = 2.4 × 10^−7^) in a mouse erythrocyte differentiation pathway [91]. And for eosinophils, of the 272 genes in the dark turquoise module, 215 met our criteria for gene significance and module membership. Again using Enrichr, we found 15 of these genes to be significantly overrepresented (FDR p = 0.003) among genes expressed by the Eol-1 cells, which is a human eosinophilic cell line [92]. Complete details for RBCs and eosinophils are provided in S6 Table.

### Case clustering

**Fig 4:**
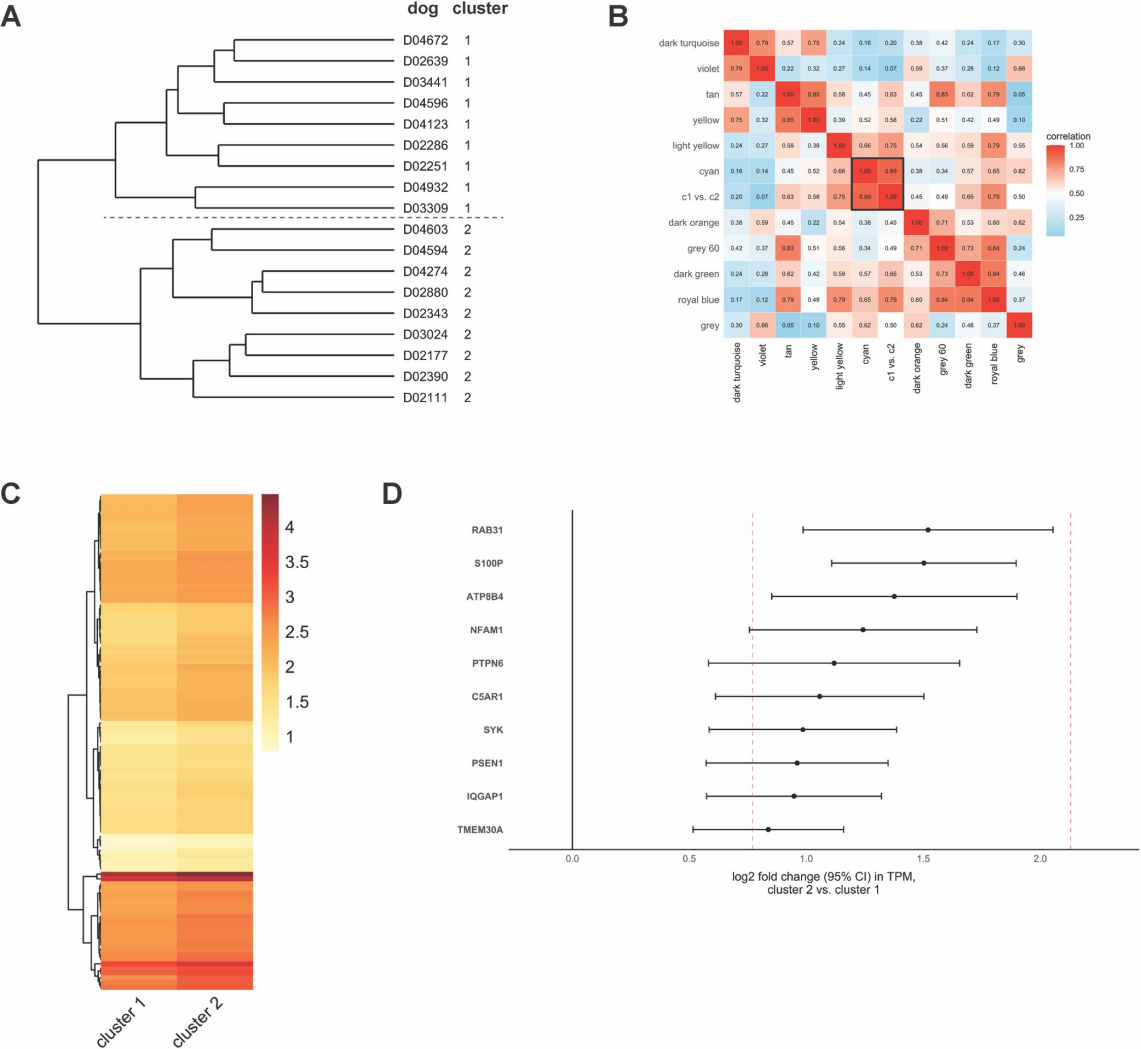
Case clustering based on gene expression data. (A) Case cluster dendrogram with primary cluster separation shown by the dashed line; dogs are labeled by cluster on the right. (B) WGCNA module adjacency matrix showing the correlation between modules, as well as the C1 vs. C2 categorical trait. The case cluster trait is highly correlated to the cyan module (r = 0.89), which is outlined in black. (C) Heatmap of 105 neutrophil-related genes distinguishing C1 from C2. Heatmap is based upon mean log_10_-scaled TPMs for each cluster. Note that the C2 genes are consistently darker (more highly expressed) than the C1 genes. (D) log_2_FC and 95% confidence interval in gene expression for the top 10 differentially expressed genes in C2 vs. C1. Dashed red lines represent the 95% confidence interval bounds in log_2_FC in neutrophil RNA based upon differences in cell count between dogs in C1 and C2.

We studied gene expression data to identify naturally occurring case clusters with potential clinical significance. Two primary case clusters were noted (C1 and C2), each containing 9 dogs, with smaller sub-clusters also present (Fig 4A). Based upon our SSE results (not shown), we focused our analysis on the primary clusters of 9 dogs each. We used these two clusters as a categorical trait in WGCNA and found that the genes driving the distinction between the two clusters were highly correlated (r = 0.89) with the cyan module (Fig 4B). Of the genes in this module, 1,352 met our criteria for gene significance, and 1,217 of these genes were able to distinguish C1 from C2 after correction for multiple testing (FDR p ≤ 0.05). We analyzed these 1,217 genes using Enrichr and found 105 of them to be highly enriched in the same three GO pathways related to neutrophil function shown in Fig 3A, left panel (FDR p = 1.6 × 10^−27^, 1.2 × 10^−27^, and 1.7 × 10^−27^ for GO pathways 0002283, 0043312, and 0002446, respectively). This finding is consistent with genes in the cyan module reflecting the inflammatory process that is observed in many IMHA patients. Of these 105 genes, all were increased in C2 vs. to C1 (Fig 4C). Among the 10 genes whose expression differed most significantly between the two clusters, the log_2_FC ranged from 0.8 to 1.5 in C2 vs. C1 (Fig 4D). This range is consistent with expected changes in RNA between the two clusters based upon differences in cell count (median [range] neutrophils for C1 8.4 × 10^3^/μL [5.4 to 17.7 × 10^3^/μL] and C2 19.0 × 10^3^/μL [10.9 to 48.2 × 10^3^/μL], Fig 4D).

### Analysis of unmapped reads

We searched for unmapped reads from microbial organisms to identify unknown infectious agents that might be associated with the onset of canine IMHA. We identified sequencing reads from four organisms whose normalized abundance met our criteria for significance in our LASSO model: feline leukemia virus (FeLV), hepacivirus C, *Mesorhizobium* spp., and *Pseudonocardia dioxanivorans* (S4 Fig). *Mesorhizobium* is a common species of bacteria found in soil [93], and *Pseudonocardia* is a bacterial species typically found as a commensal organism on certain types of ants [94]. We assumed these bacteria were environmental contaminants. Hepacivirus C is a flavivirus that causes hepatitis C in humans and is not known to infect dogs [95], however hepacivirus A is a hepacivirus C homolog that occasionally causes lung infections in dogs [96, 97]. Further analysis of the hepacivirus reads revealed that their abundance was increased in controls compared to cases (S4 Fig), however, suggesting that this organism was unlikely to be a trigger for canine IMHA.

The presence of FeLV RNA in our samples was intriguing given that certain subgroups of the virus have been shown to replicate in canine cells *in vitro* [98] and because reads were increased in cases compared to controls (S4 Fig). However, further evaluation of these reads revealed that they were likely mischaracterized, and that they were only minimally increased in cases. We assembled the unmapped, putative FeLV reads from each sample using the *de novo* assembly tool in Geneious, and then analyzed the resulting contigs using BLASTn. Across all samples, ~40% of putative FeLV reads aligned to the transcript XM_025447572.1, which is a predicted Feline Gardner-Rasheed (FGR) proto-oncogene from the *Canine lupus dingo* genome. These reads were found in both affected and unaffected dogs, and were only slightly increased in cases vs. controls, with a log_2_FC of 0.4 (95% CI 0.1–0.8).

## Discussion

In this study, we found that dogs with primary immune-mediated hemolytic anemia had increased expression of genes related to neutrophil function, coagulation, cell cycle regulation, and hematopoiesis, and decreased expression of genes related to lymphocyte function when compared to healthy age- and breed-matched controls. Some genes with notably increased expression included phospholipase scramblase 5 (PLSCR5), serpin family E member 1 (SERPINE1) which encodes for the procoagulant protein plasminogen activator inhibitor 1 (PAI-1), and genes encoding for erythrocyte membrane proteins (e.g., SLC4A1, EPB42). We did not find any genes that were differentially expressed between affected dogs that lived vs. died 30 days after diagnosis. Nor did we find any evidence of microbial RNA signatures in affected dogs that might suggest the existence of an infectious trigger for IMHA.

The most highly overexpressed gene in our study was the phospholipase scramblase PLSCR5, which had 256-fold increased expression in cases vs. controls. Phospholipase scramblases are enzymes present in all eukaryotic and prokaryotic cell membranes that assist in moving membrane phospholipids between bilayer leaflets in a non-specific, bidirectional, and energy-dependent manner [99]. Inner membrane leaflets are typically enriched in phosphatidylserine (PS), phosphatidylethanolamine, and phosphatidylinositol, whereas outer membrane leaflets are enriched in phosphatidylcholine and sphingomyelin [100]. Externalization of PS on the surface of cell membranes can trigger a variety of biological events, ranging from the initiation of the clotting cascade on platelets to programmed cell death (“eryptosis”) on erythrocytes [101]. Externalization of PS on erythrocyte membranes may help initiate blood clotting as well [88, 102], and has been implicated in the pathogenesis of hypercoagulability in dogs with IMHA [103].

While exact role of PLSCR5 has never been described in any species, its most well-studied orthologue PLSCR1 is known to play a role in modulating the innate immune response through its interactions with toll-like-receptor 9 and the adaptive immune response through its interaction with T lymphocytes [88]. Additionally, expression of PLSCR1 has been shown to be increased in monocytes of a subset of humans with systemic lupus erythematosus (SLE) at elevated risk for blood clotting, and in the monocytes of humans with anti-phospholipid syndrome [104, 105]. These links to both autoimmune disease and blood clotting, as well as the well-described role of phospholipase scramblases in externalizing procoagulant PS, suggest that further study of the role of PLSCR5 in the complex pathogenesis of canine IMHA is warranted.

We also found significantly increased expression of the gene SERPINE1 in affected dogs (17-fold overexpressed), which encodes for the enzyme PAI-1. PAI-1 is the principal inhibitor of fibrinolysis, markedly impairing clot breakdown and when elevated, causes a systemic pro-thrombotic state [106]. PAI-1 is produced by a wide variety of cells, including endothelial cells, megakaryocytes, macrophages/monocytes, and fat cells [106]. PAI-1 is also considered to be an acute phase protein whose expression is influenced by cytokines such as interleukin-1, interleukin-6 (IL-6), and TNFɑ [107–109]. The underlying reason (e.g., primary vs. reactive) or source of the increased SERPINE1 expression in IMHA cases cannot be determined from this study. However if PAI-1 levels are elevated in affected dogs, this might suggest that impaired clot breakdown plays a key role in the hypercoagulability observed in IMHA patients [103]. Therapeutics aimed at inhibiting PAI-1 may therefore be reasonable to consider in future studies once its potential role in IMHA is better understood *in vitro* [110, 111].

Additionally, we noted overexpression of many erythrocyte membrane or membrane-related proteins in affected dogs, including solute carrier family 4 member 1 (SLC4A1, which encodes for erythrocyte membrane protein band 3), spectrin beta chain (SPTB), erythrocyte membrane protein band 4.2 (EPB42), and two genes associated with the Rh blood group system (Rh associated glycoprotein [RHAG] and Rh blood group CcEe antigen [RHCE]). These genes were between 8.8–18.4-fold overexpressed in cases compared to controls. One plausible reason for increased expression of these genes may be related to the marked regenerative response observed in most of our IMHA cases. However, it is also noteworthy that some of these proteins have been implicated as autoantigens in canine IMHA [24, 112, 113] as well as AIHA and other forms of hemolytic anemias in humans [114–116]. Additionally, in humans, naturally occurring autoantibodies have been detected against erythrocyte membrane protein band 3, which are typically associated with RBC senescence and oxidative damage [117]. Further investigation into the surface expression of these proteins in erythrocyte membranes is warranted in ongoing research regarding the pathogenesis of IMHA in dogs.

The marked underexpression of lymphocyte-related genes and gene pathways was a surprising finding. We had anticipated finding overexpression of genes related to B and T cell function in affected dogs as we presumed that these cells would be hyperactivated in an immune-mediated disease. While a definitive explanation for this finding is beyond the scope of this manuscript, there are several potential reasons for this unexpected result. For example, we noted that the gene indoleamine 2,3-dioxygenase (IDO1) was 50-fold *over*expressed in affected dogs. While this gene can be upregulated in response to inflammatory cytokines [118], it has also been shown to exert profound immunosuppressive effects by catalyzing the conversion of tryptophan to N-formylkynurenine [119, 120]. This depletes tryptophan from tissues and markedly suppresses T lymphocytes which are highly sensitive to low tryptophan concentrations [121]. This would be consistent with the marked downregulation of many T lymphocyte-related pathways we observed in our analysis.

Additionally, reduced activation of T lymphocytes in patients with autoimmune disease is not without precedent. In human patients with SLE, for example, the transcription factor cAMP-responsive element modulator alpha (CREMɑ) has been shown to reduce interleukin-2 (IL-2) expression [122–126]. This, in turn, leads to decreased activity of regulatory T cells and contributes to the development of autoimmunity [122–126]. In our study, IL-2 was 8.8-fold underexpressed and CREMɑ was 1.4-fold overexpressed in affected dogs, which is consistent with this possibility. Other plausible explanations for the lymphocyte-related gene underexpression may relate to the tissue sampled in this study or in the way we estimated the change in lymphocyte RNA between cases and controls. Lymphocytes in the blood, for instance, may not be hyperactivated like lymphocytes in the liver and spleen, which is where the destruction of erythrocytes takes place in IMHA [10]. Also, our estimation of the expected change in lymphocyte RNA between cases and controls suggests that there is a 0.6-fold reduction in lymphocyte RNA in cases solely based upon differences in cell type distribution. If this estimate is too low, then this change could account for the underexpression of lymphocyte-related genes we observed. We mitigated this possibility by excluding genes with less than a four-fold change in expression between cases and controls in our pathway analysis, however. Regardless, our findings suggest that future studies investigating peripheral T cell function in dogs with IMHA are warranted to determine the reason for this observed underexpression.

To the best of our knowledge, only three prior studies have evaluated expression of genes and/or proteins in dogs with IMHA, primarily focusing on cytokines and chemokines. One study used a multiplex bead assay to evaluate serum protein levels of various cytokines, however the results are reported in groups (e.g., proinflammatory, T cell-related) rather than individually, making it difficult to compare to our findings [127]. The other two studies evaluated serum protein and gene expression levels of select cytokines [9, 128], and in comparing our findings to these results there are notable differences. For example, we found IL-2 gene expression to be markedly lower in affected dogs compared to the markedly elevated IL-2 protein levels found in both other studies. Our study and the study by Swann *et al*. were directionally consistent in CXCL8 expression (1.9-fold increased gene expression in our study, elevated serum protein levels in the Swann *et al*. paper), however the Kjelgaard-Hansen *et al*. study did not detect significant differences in this chemokine between cases and controls. There were also discrepancies in interleukin-10: Our study (gene expression) and the Swann *et al*. study (gene expression and protein concentration) did not detect differences between cases and controls, however the Kjelgaard-Hansen *et al*. study (protein concentration) did find a significant difference. With regard to IL-6, the study by Swann *et al*. reported increased serum protein levels of IL-6, however our study did not detect any IL-6 transcripts and the Kjelgaard-Hansen *et al*. study did not detect differences in protein concentrations between cases and controls. We suspect that the differences across these studies relate to what was measured in each study: we measured gene expression exclusively, whereas the other studies primarily measured protein expression. Some of the proteins that were elevated in the other studies (e.g., IL-2) may have been produced in other organs and secreted into the blood, thereby accounting for their increased levels in affected dogs. Temporal differences in the course of the disease may also play a role in explaining these differences. Further studies may elucidate additional reasons for these observed differences.

In addition to identifying differentially expressed genes and gene pathways, we used WGCNA to identify patterns in gene expression that were correlated with certain cell counts from CBC data in affected dogs. We focused our analysis on genes correlated with neutrophil count and reticulocyte count. For neutrophils, we identified 115 genes that were both associated with neutrophil count and had been previously identified in neutrophil-specific pathways. Among these 115 genes, 71 followed an expected expression pattern in which they were minimally expressed in healthy dogs and dogs with a neutrophil count in the reference range, but significantly elevated in affected dogs with a neutrophil count above the reference range. The remaining 44 genes, however, followed an unexpected expression pattern insofar as they appeared to show decreased expression in affected dogs with a normal neutrophil count (Fig 3A, center and right panels). The reason for this unexpected expression pattern cannot be determined from this exploratory study, however one possible reason is that affected dogs with a normal neutrophil count have a different underlying etiology for their disease which does not trigger an inflammatory response. Another possibility may be temporal, and that these genes might eventually become activated at later stages in the disease process. Further studies are warranted to more fully explore the nature of the inflammatory process in dogs with IMHA.

For reticulocytes, we identified 185 genes that were both associated with reticulocyte count and had been previously identified in pathways associated with early erythroid development. Expression of these genes was minimal in unaffected dogs but increased in clusters as reticulocyte counts of affected dogs increased. These findings are consistent with an orderly progression of the regenerative response, and for the first time identify genes in dogs that may be activated at different stages of process. Interestingly, a cluster of genes appears to be activated only in “super regenerators” with reticulocyte counts >300 × 10^6^/μL (Fig 3B, center and right panels). The significance of this finding is unclear, and as with the neutrophil-related genes, may relate to the time in the disease process at which these dogs were sampled. Future studies may help explain the reasons for differences in gene expression relative to the regenerative response that we observed in affected dogs.

Based upon gene expression data, we identified two primary case clusters in affected dogs that are highly driven by neutrophil-related genes and associated with the neutrophil cell count. The significance of this finding is unclear. Because we sampled dogs at a single time point, it is possible that this clustering is purely temporal and that dogs with a minimal inflammatory response at the beginning of their disease course will eventually develop an elevated neutrophil count. Another possibility, as we suggested earlier, is that neutrophil gene expression and neutrophil count may be a marker of a different underlying disease etiology. Further studies that evaluate changes in neutrophil count over time in affected dogs may begin to help resolve this important question.

Using unmapped transcripts, we searched for evidence of an infectious organism that might serve as a trigger for IMHA in dogs, however we were unable to find any such evidence in our dataset. The possibility of an infectious trigger for canine IMHA has been proposed by several authors [127, 129, 130], and our findings do not exclude this possibility. For example, our sample collection and library preparation methods focused exclusively on total RNA, and therefore may be more likely to identify RNA-based viruses or organisms undergoing active replication and transcription. Also, there may be geographic differences in the likelihood of finding potential organisms that trigger IMHA as suggested by others [129], and these organisms may not be present in the geographic areas from which our samples originated. Finally, it is possible that small amounts of non-canine RNA may be below the limits of detection of our analysis, or that no single infectious organism is common to all cases. A more comprehensive study using next-generation sequencing methods specifically aimed at identifying infectious organisms is warranted to resolve this ongoing question.

There are several limitations to this study. First, it is important to emphasize that we used whole blood as a source of RNA for this study. Whole blood contains a mixed cell population, and the proportions of these cells differ significantly between dogs with and without IMHA (S1 Fig). This, in turn, led to differences in the proportion of RNA originating from each cell type between cases and controls (S1 Fig). We attempted to estimate these changes using cell count data from each animal as well as published values for each cell type, and found that for most relevant of cell types, differences in cell counts account for a 0.6–4.4-fold change in gene expression between cases and controls. This value is only an estimate based upon published RNA mass by cell type in other species, as canine data is not available. We contemplated methods used by others to normalize for differences in cell counts when performing RNA sequencing on whole blood [131, 132], especially using blood cell counts as covariates when testing for DEGs. After careful consideration, however, we opted against this method as it would adjust expression of all transcripts for all cell types, despite some transcripts only originating from a single cell type (e.g., a neutrophil-specific transcript would be adjusted for differences in reticulocyte and platelet count). Our approach is similar to others, where no adjustments for cell type were made [29, 133, 134]. Instead, we opted to estimate the extent to which our expression data would be impacted by changes in cell counts, and limit our analysis to those genes beyond this estimated range. Future studies examining gene expression in individual cell types (e.g., using cell sorting or single-cell RNA sequencing) are undoubtedly warranted, however this was beyond the scope of this study.

Second, we must acknowledge that some of the changes in gene expression we observed in IMHA cases could be common in any dog with a regenerative anemia or an inflammatory response. It is therefore impossible to ascribe all of the changes we observed as being unique to IMHA patients. In fact, many of the changes in gene expression we documented are correlated with changes in underlying blood cell count (e.g., Fig 3B). Further studies evaluating gene expression in the blood of dogs with regenerative anemias due to other causes (e.g., acute blood loss) or with inflammatory responses due to other causes (e.g., septic abdomen) could help pinpoint changes in gene expression that are unique to IMHA patients. Collecting these samples was beyond the scope of this study, but should be considered as part of any going work in this area.

Additionally, because we used whole blood for this study, we will invariably exclude changes in gene expression in the liver and spleen of affected dogs, which is where the majority of erythrocyte destruction takes place in IMHA. Hence, our findings to not provide a complete picture of gene expression changes that occur in the disease. Future studies that include liver and/or spleen samples in affected dogs may be useful in overcoming this limitation. And finally, we must emphasize that RNA sequencing studies are inherently exploratory, and cannot explain the reasons for observed changes in gene expression. However, studies such as ours provide important insights regarding areas of future research that might ultimately answer these fundamental questions.

In summary, we found that dogs with IMHA had increased expression of genes related to neutrophil function, coagulation, cell cycle regulation, and hematopoiesis, and decreased expression of genes related to lymphocyte function. We also identified genes that are associated with the development of the inflammatory and regenerative RBC response in affected dogs. Future studies, particularly studies examining changes in gene expression in individual cell types, are warranted to confirm these findings, along with studies to further evaluate the role of highly differentially expressed genes (e.g., phospholipase scramblases) in the pathogenesis of canine IMHA.

## Supporting information

S1 Fig Estimated effects of cell type distribution on RNA abundance. (A) Box and whiskers plots showing the abundance of cell types and estimated cellular RNA between cases and controls. (B) Stacked bar chart scaled to 100% showing the average fraction of cell types (left) and estimated cellular RNA (right) between cases and controls. (C) Estimated log_2_ fold-change in fraction of RNA per μL between cases and controls by cell type, with 95% confidence intervals shown as dashed red vertical lines.
(TIF)

S2 Fig Principal components analysis. PCA analysis using VST-transformed read counts identified three sample outliers (all cases). Samples are coded as follows: red, affected/included; blue, unaffected/included; black, affected/excluded.
(TIFF)

S3 Fig WGCNA modules and trait matrix. (A) Cluster dendrogram showing clustering of genes and creation of modules; (B) Trait matrix showing the relationship between 11 modules and the counts of 7 blood cell phenotypes. Module names/colors are shown on the left, and gene counts within each module are in parenthesis. Module significance is shown on the right of the figure; 7 modules are significant. Shading represents the correlation between each trait and module, with positively correlated values shown in red and negatively correlated values shown in green. Numbers within each tile indicate the p-value for the significance of each module-trait relationship. Tiles outlined in black represent those module-trait relationships that were explored further based upon their relevance to canine IMHA.
(TIF)

S4 Fig Analysis of unmapped reads. (A) LASSO model output showing organisms whose mapped reads were top predictors of IMHA disease status; gray bar at 0.6 represents our frequency cutoff for significance. (B) Normalized read counts (counts per million) mapped to feline leukemia virus, cases vs. controls. (C) Normalized read counts (counts per million) mapped to hepacivirus C, cases vs. controls.
(TIF)

S1 Table PCR primers. Primers for qPCR of T cell pathway genes and housekeeping genes.
(XLSX)

S2 Table Demographic and laboratory data for enrolled cases and controls. Data for cases and controls is provided on separate tabs.
(XLSX)

S3 Table RNA sequencing quality metrics. (XLSX)

S4 Table Expressed transcripts and differentially expressed genes. DESeq2 output for expressed transcripts (14,055) and DEGs (966); data is provided on separate tabs.
(XLSX)

S5 Table GSEA over- and underexpressed pathways. Data for over- and underexpressed pathways is shown on separate tabs.
(XLSX)

S6 Table WGCNA module-trait gene-level data. Module-trait gene-level data for neutrophils, reticulocytes, RBCs, and eosinophils. Each tab contains the genes within each colored module, module membership, gene significance, associated p-values, and an indication of whether a gene was a member of the pathway referenced in the manuscript. For neutrophils and reticulocytes, cluster-level information is also provided for pathway. Each tab also contains a plot of module membership vs. gene significance for all genes within that module.
(XLSX)
