# Supplementary figures and images for: RNA sequencing of whole blood in dogs with primary immune-mediated hemolytic anemia (IMHA) reveals novel insights into disease pathogenesis
Source: PLoS One. 2020 Oct 22;15(10):e0240975. doi: 10.1371/journal.pone.0240975 (PMC7580939; doi:10.1371/journal.pone.0240975)

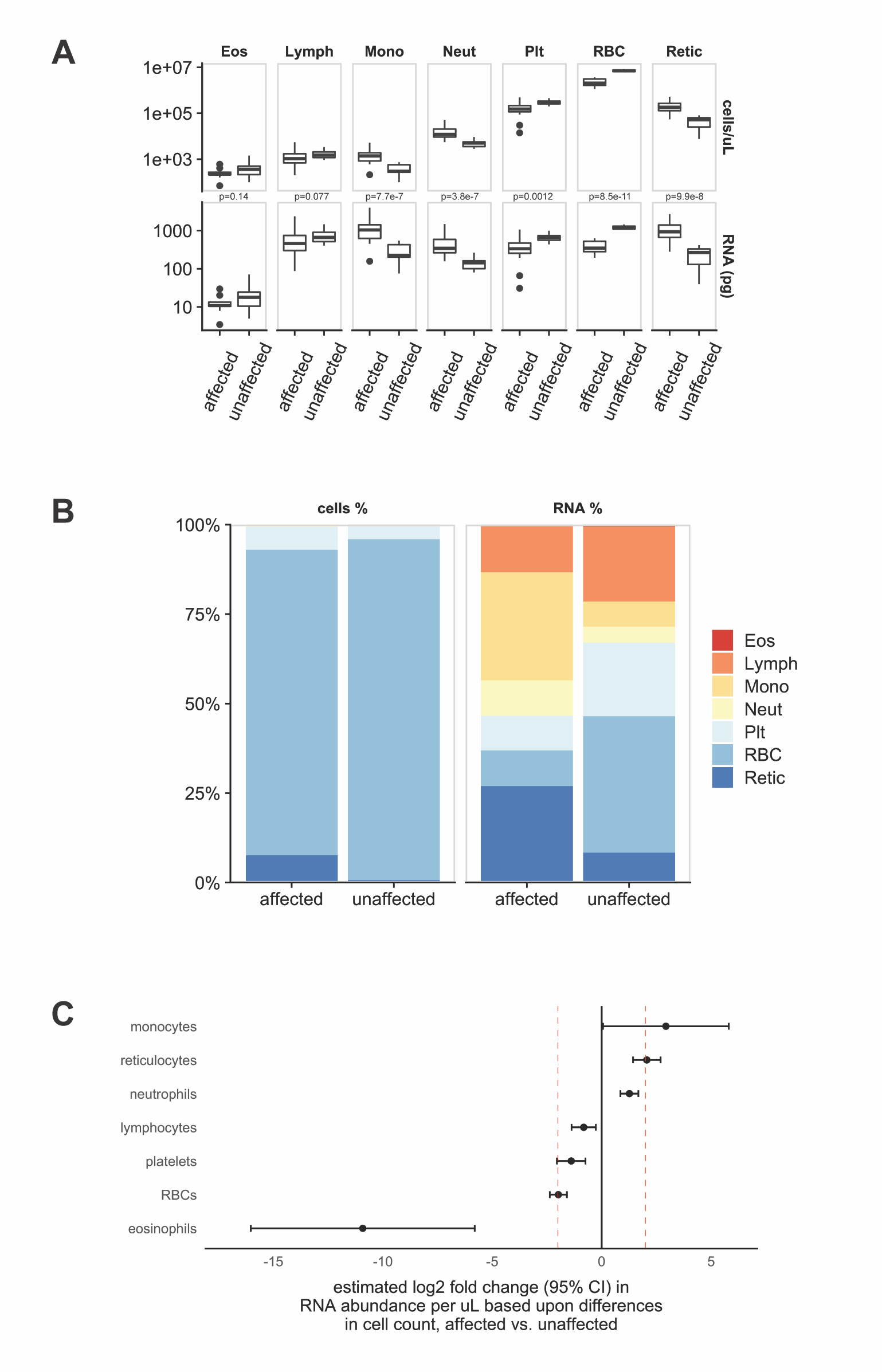

Supplement: S1 Fig — (A) Box and whiskers plots showing the abundance of cell types and estimated cellular RNA between cases and controls. (B) Stacked bar chart scaled to 100% showing the average fraction of cell types (left) and estimated cellular RNA (right) between cases and controls. (C) Estimated log2 fold-change in fraction of RNA per μL between cases and controls by cell type, with 95% confidence intervals shown as dashed red vertical lines. (TIF) [file pone.0240975.s001.tif]

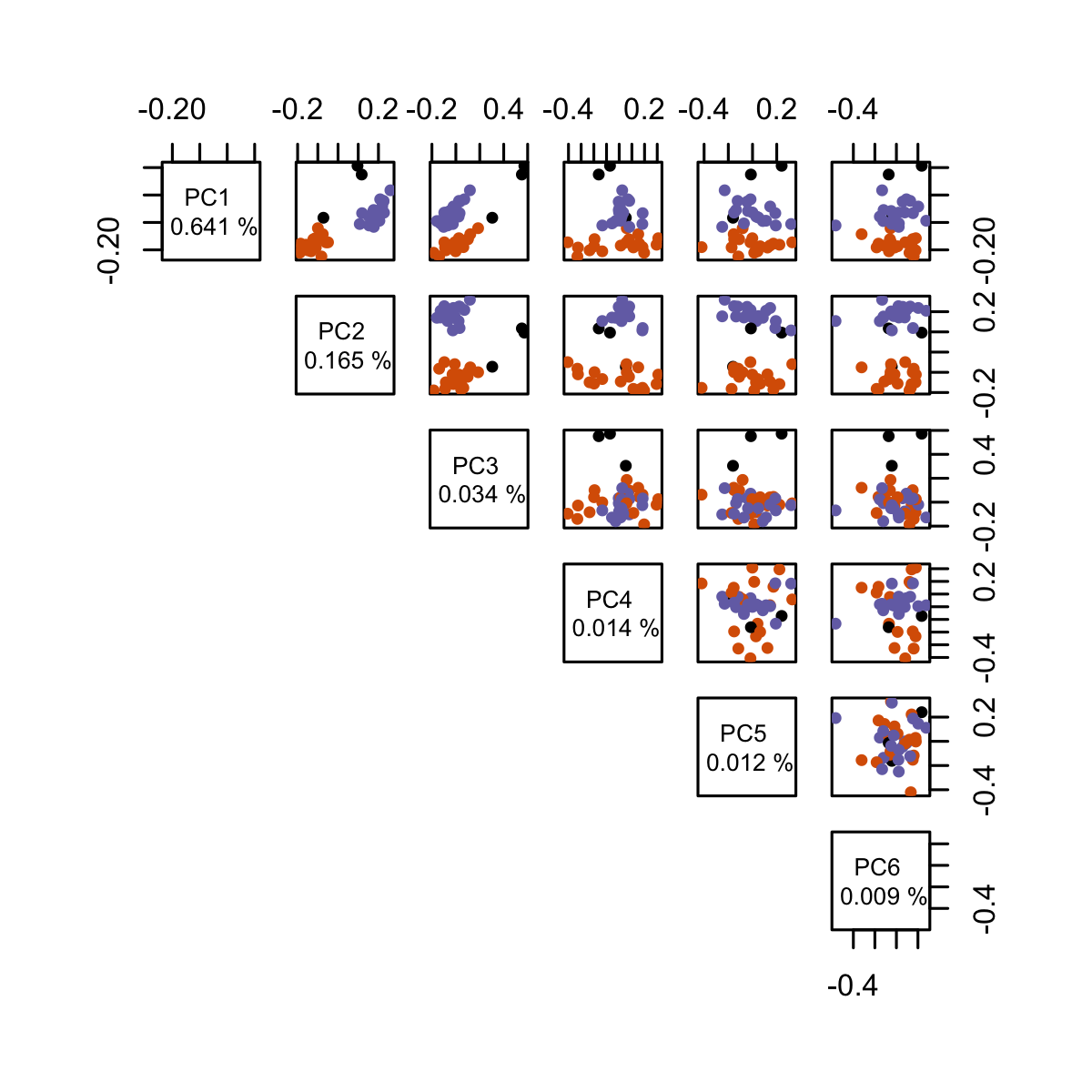

Supplement: S2 Fig — PCA analysis using VST-transformed read counts identified three sample outliers (all cases). Samples are coded as follows: red, affected/included; blue, unaffected/included; black, affected/excluded. (TIFF) [file pone.0240975.s002.tiff]

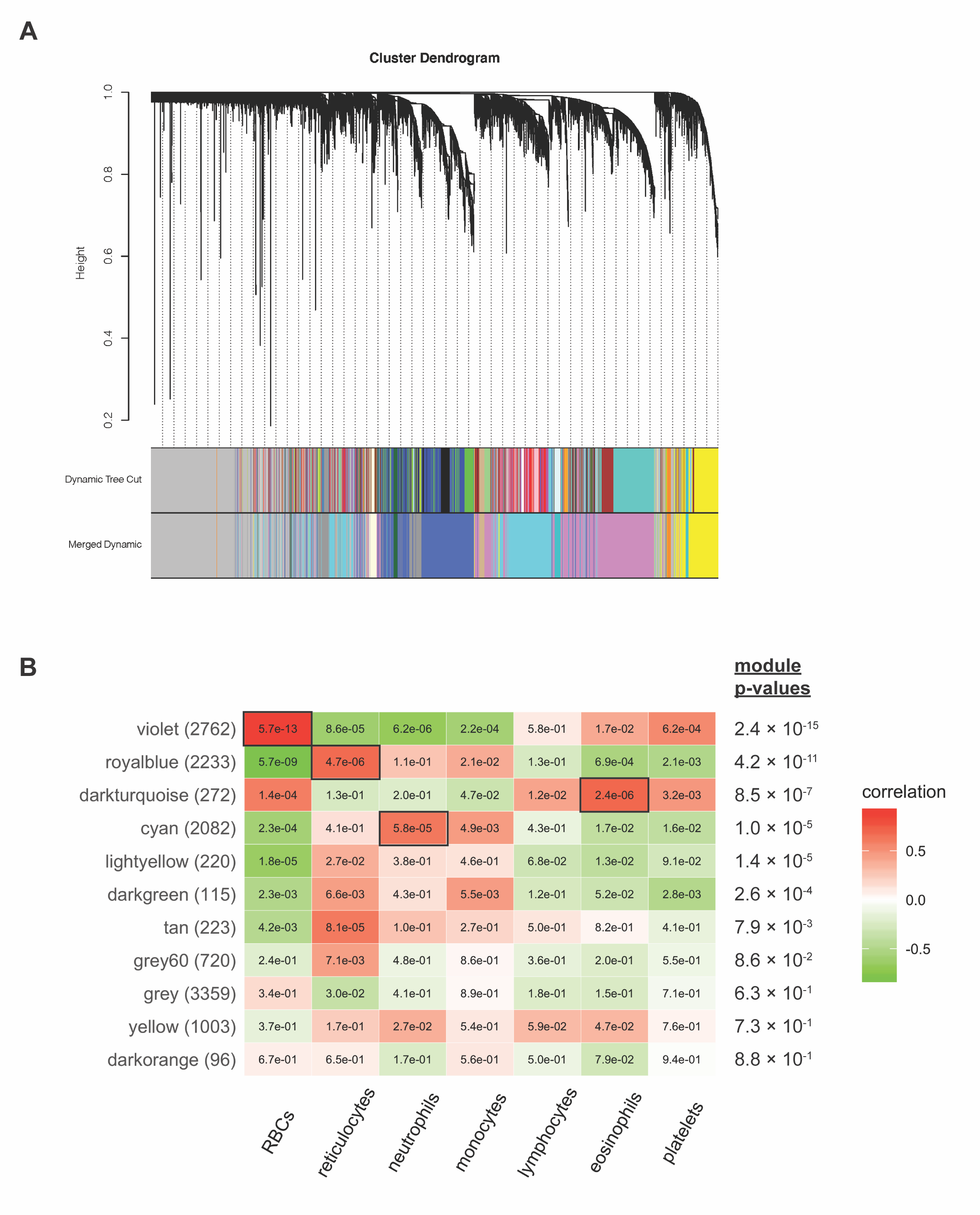

Supplement: S3 Fig — (A) Cluster dendrogram showing clustering of genes and creation of modules; (B) Trait matrix showing the relationship between 11 modules and the counts of 7 blood cell phenotypes. Module names/colors are shown on the left, and gene counts within each module are in parenthesis. Module significance is shown on the right of the figure; 7 modules are significant. Shading represents the correlation between each trait and module, with positively correlated values shown in red and negatively correlated values shown in green. Numbers within each tile indicate the p-value for the significance of each module-trait relationship. Tiles outlined in black represent those module-trait relationships that were explored further based upon their relevance to canine IMHA. (TIF) [file pone.0240975.s003.tif]

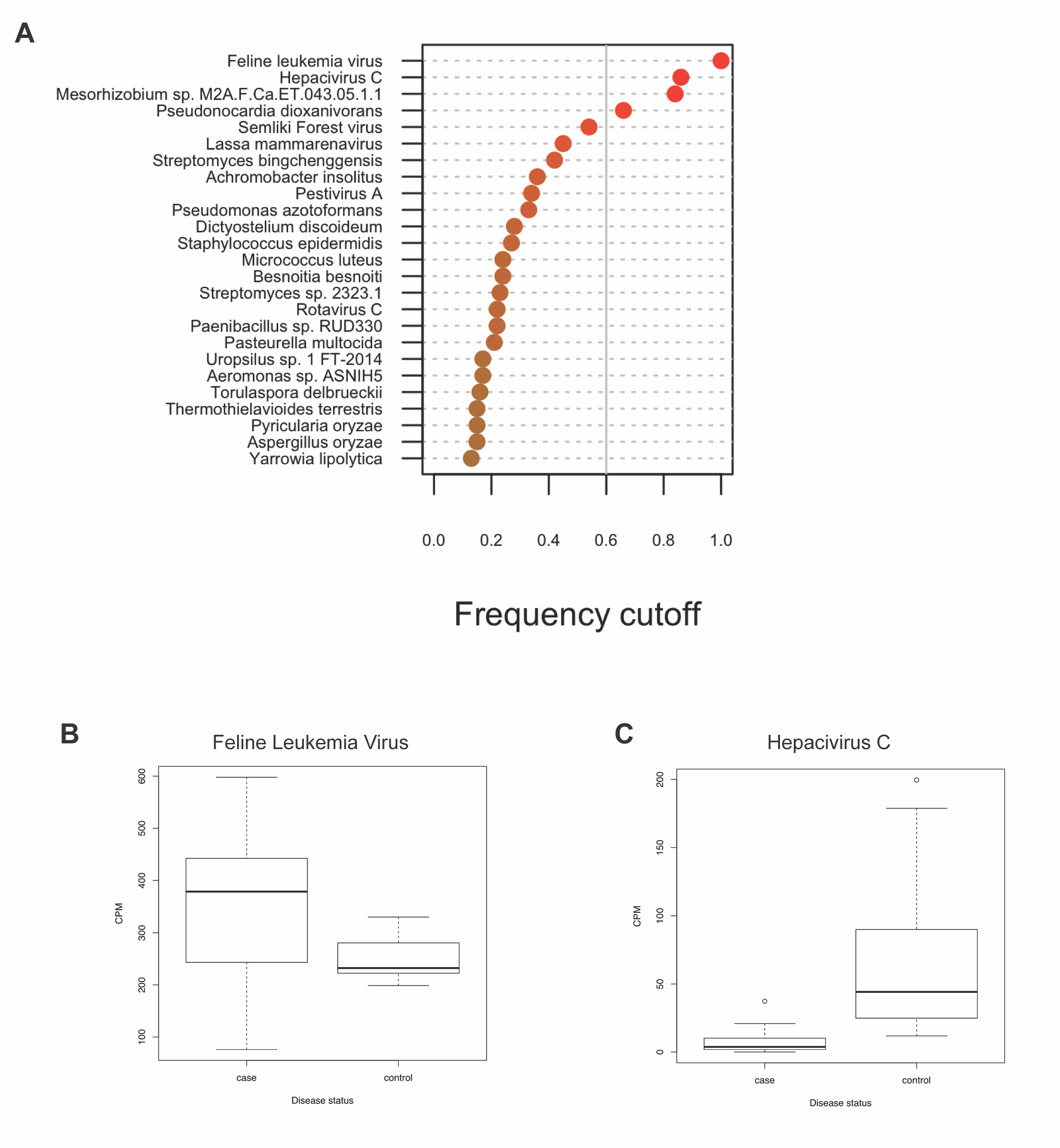

Supplement: S4 Fig — (A) LASSO model output showing organisms whose mapped reads were top predictors of IMHA disease status; gray bar at 0.6 represents our frequency cutoff for significance. (B) Normalized read counts (counts per million) mapped to feline leukemia virus, cases vs. controls. (C) Normalized read counts (counts per million) mapped to hepacivirus C, cases vs. controls. (TIF) [file pone.0240975.s004.tif]
